# Prevalence of molecular markers associated with drug resistance of *Plasmodium vivax* isolates in Western Yunnan Province, China

**DOI:** 10.1186/s12879-020-05032-4

**Published:** 2020-04-25

**Authors:** Xiaoxiao Wang, Wei Ruan, Shuisen Zhou, Xinyu Feng, He Yan, Fang Huang

**Affiliations:** 1 National Institute of Parasitic Diseases, Chinese Center for Disease Control and Prevention, Key Laboratory of Parasite and Vector Biology, MOH, and WHO Collaborating Centre for Malaria, Schistosomiasis and Filariasis, Shanghai, People’s Republic of China; 2 Zhejiang Provincial Center for Disease Control and Prevention, Zhejiang, People’s Republic of China

## Abstract

**Background:**

*Plasmodium vivax* is the most widely distributed malaria parasite, and its drug resistance poses unique challenges to malaria elimination. The Greater Mekong Subregion (GMS) is known as the global epicenter of multidrug resistance. Surveillance of molecular markers associated with drug resistance in this area will help to inform drug policy.

**Methods:**

Dry blood spots from 58 patients out of 109 with *P. vivax* infection between 2017, December and 2019, March were obtained from Yingjiang County, Yunnan Province, along the China–Myanmar border. *Pvdhfr, Pvdhps, Pvmdr1* and *Pvcrt-o* were amplified and sequenced to assess gene mutations. The polymorphism and prevalence of these molecular markers were analyzed.

**Results:**

Mutations in *Pvdhfr* at codons 57, 58, 61, 99 and 117 were detected in 27.59, 48.28, 27.59, 32.76 and 48.28% of the isolates, respectively. Single mutant haplotype (I_13_F_57_S_58_T_61_**S**_99_S_117_I_173_) was the most frequent (29.31%, 17/58), followed by double mutant haplotype (20.69%, 12/58). Of three types of tandem repeat variations of *Pvdhfr*, deletion type was the most common. *Pvdhps* showed a lower prevalence among mutation genotypes. Single mutant was dominant and accounted for 34.48% (20/58). Prevalence of *Pvmdr1* mutations at codons 958 and 1076 were 100.00% and 84.48%, respectively. The proportion of double and single mutant types was 84.48% (49/58) and 15.52% (9/58), respectively. Eleven samples (18.97%, 11/58) showed K10 “AAG” insertion in chloroquine resistance transporter gene *Pvcrt-o*.

**Conclusions:**

There was moderate diversity of molecular patterns of resistance markers of *Pvdhfr*, *Pvdhps*, *Pvmdr1* and *Pvcrt-o* in imported *P. vivax* cases to Yingjiang county in Western Yunnan, along the China–Myanmar border. Prevalence and molecular pattern of candidate drug resistance markers *Pvdhfr*, *Pvdhps*, *Pvmdr1* and *Pvcrt-o* were demonstrated in this current study, which would help to update drug policy.

## Background

*Plasmodium vivax* is the most widely distributed malaria parasite, and although it causes less significant morbidity and mortality than *Plasmodium falciparum* does, it poses unique challenges in many countries [1]. In 2017, it was estimated to be responsible for 7.5 million cases globally, and nearly 56% in Southeast Asia [2]. Recently, there has been a massive reduction in malaria cases and deaths in the Greater Mekong Subregion (GMS), which comprises Cambodia, Yunnan Province of China, Lao People’s Democratic Republic, Myanmar, Thailand and Vietnam. However, GMS has been the global epicenter of multidrug resistance. Resistance emerged to chloroquine (CQ) in the 1960s, sulfadoxine–pyrimethamine (SP) in the 1970s, mefloquine in the late 1990s, and artemisinin in 2008, and then spread progressively throughout other malaria-endemic areas [3–6]. This has raised concern from the World Health Organization (WHO) and local health authorities [2, 7, 8]. Malaria transmission in international border areas is usually confounded by population mobility and distinct chemotherapy policies and antimalarial strategies. The China–Myanmar border, as part of the GMS, included 18 counties of Yunnan Province. Although no indigenous cases have been identified in Yunnan Province since 2017, *P. vivax* remains a challenge, with increasing evidence of abundant vector species richness and diversity, high malaria vulnerability resulting from mobile population, as well as drug resistance [9, 10]. In Myanmar, the proportion of malaria cases caused by *P. vivax* has increased steadily since 2012 [11].

CQ was first produced in 1934 and quickly proved to be one of the most successful and important antimalarial agents [12]. Nevertheless, the heavy use of CQ throughout subsequent decades eventually led to drug resistance. *P. falciparum* developed resistance in various areas since the 1950s, but drug-resistant *P. vivax* was not reported until the 1980s in Indonesia and Papua New Guinea [12, 13]. To date, CQ-resistant *P. vivax* has been confirmed in more than 10 countries, including Myanmar and China [14]. There has been a long history of successful application of SP in combating malaria due to its safety, good tolerance and long-lasting activity [15]. In China, as a component of the two combination regimens, pyrimethamine was widely used for malaria prophylaxis between the mid-1960s and early 1990s [16]. By now, SP is recommended by WHO as one of the partner drugs for treatment of *P. falciparum* in the GMS, as well as intermittent preventive treatment for infants, children and pregnant women [2, 15]. Although SP is rarely used to treat *P. vivax* infection, the parasite is still under SP selection pressure, especially in endemic regions where co-infection with *P. vivax* and *P. falciparum* is common.

Compared with *P. falciparum*, it is more difficult to determine the underlying mechanisms of antimalarial drug resistance of *P. vivax* because there is no proper in vitro cultivation system for *P. vivax*. This means that the molecular mechanism of *P. vivax* resistance remains to be established. Several studies suggest that it involves multigenic loci, such as CQ resistance marker *Pvcrt-o*; multidrug resistance marker *Pvmdr1*; and antifolate resistance markers *Pvdhps* and *Pvdhfr*, which are conferred from homologous genes in *P. falciparum* [11, 17]*.* Data for molecular markers associated with drug resistance would be beneficial in addressing the resistant parasite. Few studies to date have defined the molecular epidemiology of *P. vivax* resistance markers on the China–Myanmar Border [18, 19]. Here, we report the prevalence of molecular markers of drug resistance in *P. vivax* to facilitate appropriate drug policy in this region.

## Methods

### Study site

Yingjiang (Longitude 97°31′ ~ 98°16′,Latitude 24°24′ ~ 25°20′) is one of the 18 counties along the China–Myanmar border, located west of Yunnan Province. It was selected as the study site due to its long borderline with Kachin State, Myanmar and being well documented as an epidemic area of resistant *P. falciparum* [18]*.* The land area of Yingjiang County is 4429 km^2^ and the local population was 316, 990 by 2015. It is located in the subtropical monsoon climate zones with an average annual temperature of 22.7 °C and annual rainfall of 2.65 m. Migration, plantation and logging activities are frequent at the border [18]. *Anopheles minimus* is reported to be the dominant species of mosquito [20]. Ninety-three malaria cases were reported in Yingjiang County in 2016 and 2017, respectively, and there were 103 cases in 2018. *P. vivax* was the dominant parasite and all the cases of malaria were imported after May, 2016.

### Sample collection and DNA extraction

Isolates were obtained from 58 out of 109 confirmed *P. vivax* infected patients from December 2017 to March 2019 in Yingjiang County. All the infections were diagnosed and reported by hospitals or clinics in Yingjiang County. Yingjiang County Center for Disease Control and Prevention carried out epidemiological investigation of each patient. They were double-checked for species by PCR in Yunnan Institute of Parasitic Diseases. All the patients, according to epidemiological history, were imported from Laiza, Myanmar, which was along the China–Myanmar border. Thick and thin blood smears coupled with standard microscopy techniques were used to identify parasite species, then PCR was used to double check and confirm species. Approximately 200 μL of finger-prick blood was obtained from each patient before treatment and spotted on Whatman 3 MM filter paper (10 cm × 7 cm, Cat. No. 3030–866) and air dried. The dried blood spot was about 6 mm for diameter. They were stored in small plastic zip lock bags with desiccants at − 20 °C before parasite genomic DNA extraction. QIAamp DNA Mini kit (Qiagen Inc., Hilden, Germany) was used to extract genomic DNA following the dried blood spot protocol.

### DNA amplification and sequencing

Multiple molecular markers, *Pvcrt-o*, *Pvmdr1*, *Pvdhps* and *Pvdhfr*, suspected conferring drug resistance on *P. vivax*, were detected*. Pvcrt-o* was amplified by regular PCR and *Pvmdr1*, *Pvdhps* and *Pvdhfr* by nested PCR, as previously described, with some modification [17, 21]*.* Oligonucleotide primers and cycling conditions are listed in additional file (see Additional file: Table S1). A final 25-μL reaction volume was performed, of which 1 μL template genomic DNA was used in primary amplification reactions, and 1 μL primary reaction products in the second round of amplification in the case of nested PCRs. Amplification products were sequenced by Sangon Biotech Co. Ltd. (Shanghai, China).

### Data analysis

Nucleotide and amino acid sequences of *Pvcrt-o*, *Pvmdr1*, *Pvdhps* and *Pvdhfr* were aligned and compared with reference sequences from NCBI database by Mega version 7.0.26 (https://www.megasoftware.net/). Accession numbers for reference sequences were: *Pvcrt-o* (AF314649), *Pvmdr1*(AY618622), *Pvdhps* (XM001617159) and *Pvdhfr*(X98123)*.* A database was constructed by Microsoft Excel 2017, and descriptive statistical analysis was performed with SPSS Statistics for Windows version 21.0 (IBM Corp., Armonk, NY, USA). Categorial data were summarized by percentage, quantitative variables were expressed as median.

## Results

### General information

We collected data from 58 patients (40 males,68.97%; 28 females,31.03%) with *P. vivax* infections between 2017 and 2019, of which, the majority (89.66%, 52/58) were collected in 2018. The median (range) age of the 58 patients was 34.5 (3–69) years. Nine (15.52%) patients had a history of malaria. Most patients lived (4/58,6.90%), studied (1.72%,1/58) or worked (planting,50.00%,29/58; trading, 6.90%,4/58) in Myanmar, whereas 20 patients were infected when they visited relatives or friends (27.59%,16/58), or during business trips (6.90%,4/58) in Myanmar (Table 1).

**Table 1:** General information of *P. vivax* infections

General information	Number (%)
Year	
2017	3 (5.17)
2018	52 (89.66)
2019	3 (5.17)
Gender	
Male	40 (68.97)
Female	18 (31.03)
Age	
Range	3 ~ 69 yr
Median	34.5 yr
History of malaria infection	
Yes	9 (15.52)
No	49 (84.48)
Activities in Myanmar	
Planting	29 (50.00)
Visiting relatives or friends	16 (27.59)
Business trip	4 (6.90)
Trading	4 (6.90)
Living	4 (6.90)
Studying	1 (1.72)

### Prevalence and patterns of *Pvdhfr* mutations

**Fig. 1:**
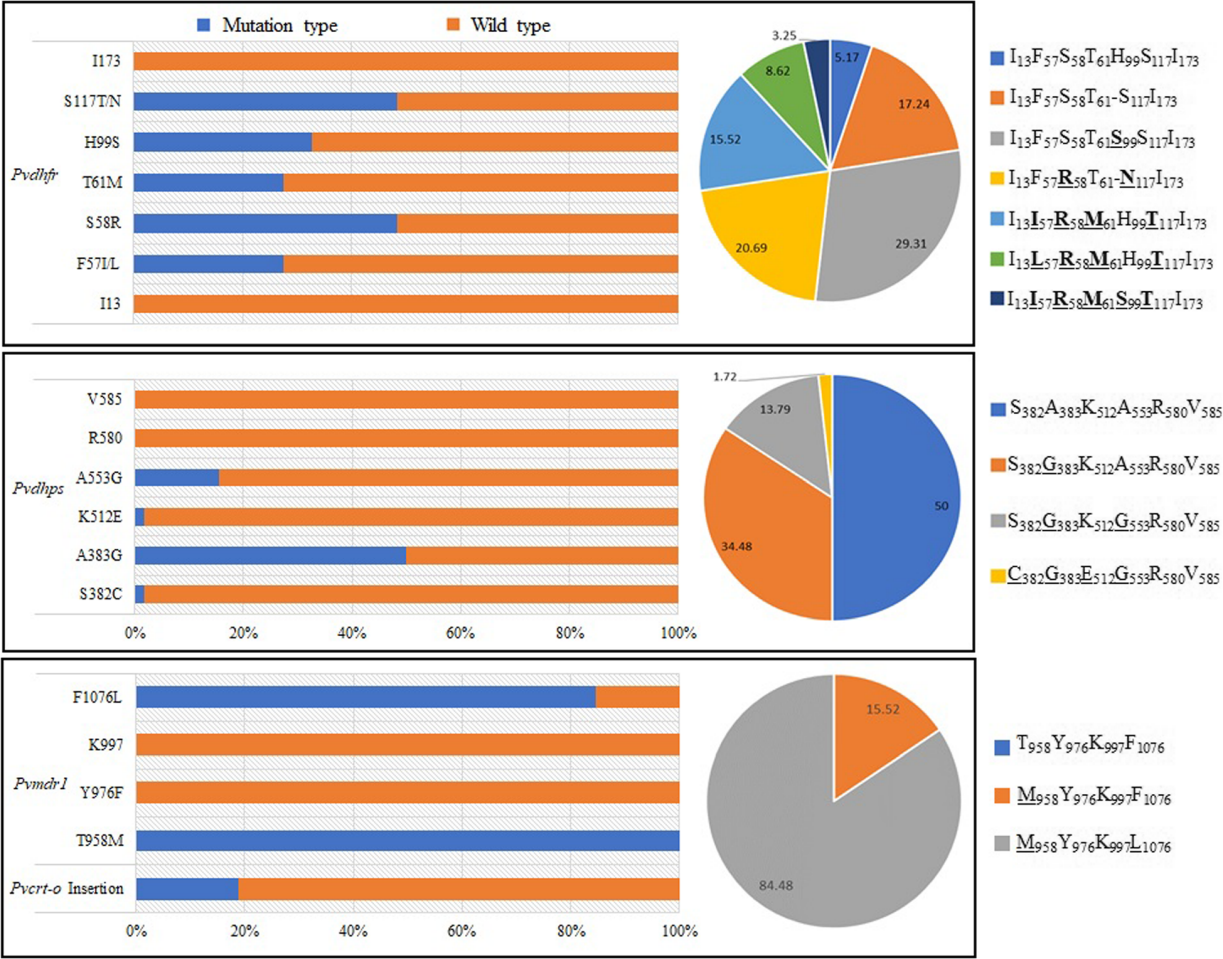
Prevalence of point mutations and haplotypes of *Pvcrt-O*, *Pvmdr1*, *Pvdhps* and *Pvdhfr* in *P. vivax* isolates

Mutations in *Pvdhfr* at codons 57, 58, 61, 99 and 117 were detected in 27.59, 48.28, 27.59, 32.76 and 48.28% of isolates, respectively. No mutations were found at position 13 or 173 (Table 2). Analysis of *Pvdhfr* haplotype revealed that prevalence of mutant types was present at high levels (Table 3, Fig. 1). Both single and multiple mutant *Pvdhfr* (double, quadruple and quintuple) were found. Single mutant haplotype (I_13_F_57_S_58_T_61_**S**_99_S_117_I_173_) was the most frequent (29.31%,17/58), followed by double mutant haplotype (20.69%,12/58). Quadruple mutant haplotypes, exhibiting two distinct patterns, were also found in 14 isolates, and the pattern I_13_I_57_**R**_58_**M**_61_H_99_**T**_117_I_173_ was more common. Quintuple mutant I_13_**I**_57_**R**_58_**M**_61_**S**_99_**T**_117_I_173_ was detected in two isolates. Notably, two genotypes were detected at codons 57 and 117. Specifically, F57I and F57L at position 57, were observed in 11(18.97%) and 5(8.62%)isolates, respectively, and the frequency for S117T and S117N was 16(27.59%) and 12(20.69%), respectively.

**Table 2:** Prevalence of point mutations at specific positions in *Pvcrt-o, Pvmdr1, Pvdhps* and *Pvdhfr* of *P. vivax* isolates

Genes	Mutation at codon	Number (%)
*Pvdhfr*		
	13	0
	57	16 (27.59)
	58	28 (48.28)
	61	16 (27.59)
	99*	19 (32.76)
	117	28 (48.28)
	173	0
*Pvdhps*		
	382	1 (1.72)
	383	29 (50.00)
	512	1 (1.72)
	553	9 (15.52)
	580	0
	585	0
*Pvmdr1*		
	958	58 (100.00)
	976	0
	997	0
	1076	49 (84.48)
*Pvcrt-o*		
	K10 insertion	11 (18.97)

^a^: Deletion type was not included

**Table 3:** Prevalence of haplotypes of *Pvcrt-o, Pvmdr1, Pvdhps* and *Pvdhfr* in *P. vivax* isolates

Genes	Haplotype	Codon^a^	Number (%)
*Pvdhfr*			
	Wild type	I_13_F_57_S_58_T_61_H_99_S_117_I_173_	3 (5.17)
	Mutant type		55 (94.83)
	Mutant tandem repeat	I_13_F_57_S_58_T_61_-S_117_I_173_	10 (17.24)
	Single mutant	I_13_F_57_S_58_T_61_**S**_99_S_117_I_173_	17 (29.31)
	Double mutant and tandem repeat	I_13_F_57_**R**_58_T_61_-**N**_117_I_173_	12 (20.69)
	Quadruple mutant(a)	I_13_**I**_57_**R**_58_**M**_61_H_99_**T**_117_I_173_	9 (15.52)
	Quadruple mutant(b)	I_13_**L**_57_**R**_58_**M**_61_H_99_**T**_117_I_173_	5 (8.62)
	Quintuple mutant	I_13_**I**_57_**R**_58_**M**_61_**S**_99_**T**_117_I_173_	2 (3.45)
*Pvdhps*			
	Wild type	S_382_A_383_K_512_A_553_R_580_V_585_	29 (50.00)
	Single mutant	S_382_**G**_383_K_512_A_553_R_580_V_585_	20 (34.48)
	Double mutant	S_382_**G**_383_K_512_**G**_553_R_580_V_585_	8 (13.79)
	Quadruple mutant	**C**_382_**G**_383_**E**_512_**G**_553_R_580_V_585_	1 (1.72)
*Pvmdr1*			
	Wild type	T_958_Y_976_K_997_F_1076_	0
	Single mutant	**M**_958_Y_976_K_997_F_1076_	9 (15.52)
	Double mutant	**M**_958_Y_976_K_997_**L**_1076_	49 (84.48)
*Pvcrt-o*			
	Wild type		47 (81.03)
	Mutant (“AAG” insertion)		11 (18.97)

a:Mutant amino acids are shown in bold

**Fig. 2:**
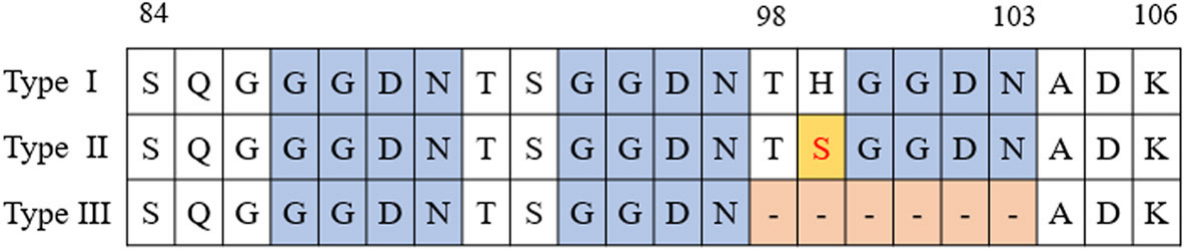
Sequence alignment of tandem repeat region between amino acid positions 84 and 106 in *Pvdhfr* gene. Dashes represent tandem repeat deletion between 98 and 103. Blue indicates the tandem repeat. Orange denotes the mutant at codon 99

Three types of tandem repeat variations were found in *Pvdhfr.* Type I was the same as the reference strain (accession number X98123), whereas type II showed mutant allele H99S, and type III exhibited a deletion of 18 nucleotides (ACACACGGTGGTGACAAC, translated into THGGDN) between amino acid positions 98 and 103 (Fig. 2). Type III was the most common, accounting for 37.93% (22/58), followed by Type II which was observed in 19 (32.76%) isolates. In addition, more than half of Type III isolates (12/22, 54.55%) also carried S58R and S117N mutations.

### Prevalence and patterns of *Pvdhps* mutations

All 58 samples were successfully amplified for *Pvdhps*. Compared with *Pvdhfr*, *Pvdhps* showed a relatively lower prevalence of mutation genotypes. Minority of isolates carried mutations at codons 382 (1.72%, 1/58), 512 (1.72%, 1/58) and 553 (15.52%, 9/58) (Fig. 2). Mutation at position 383 was detected in half of the isolates. Among the mutant types, single mutant was dominant and accounted for 34.48% (20/58). Double mutant S_382_**G**_383_K_512_**G**_553_R_580_V_585_ was less frequent (13.79%, 8/58). Quadruple mutant **C**_382_**G**_383_**E**_512_**G**_553_R_580_V_585_ was only found in one *P. vivax* isolate. S382C and K512E were rarely observed in previous studies.

### Prevalence and patterns of *Pvmdr1* and *Pvcrt-o* mutations

Prevalence of mutations at codons *Pvmdr1* 958 and 1076 was 100.00 and 84.48%, respectively. No single nucleotide polymorphism was present at either codon 976 or 997. Analysis of *Pvmdr1* haplotype prevalence showed that all the isolates were mutant type. In particular, double mutant type predominated (84.48%, 49/58). Single mutant was found in nine isolates (15.52%, 9/58). Eleven samples (18.97%, 11/58) showed K10 “AAG” insertion in CQ resistance transporter gene *Pvcrt-o* (Fig. 2). A combined analysis of all mutations in 58 samples revealed 25 different haplotypes (see Additional file: Table S2).

## Discussion

Drug resistance is of great concern for malaria control and prevention, especially in GMS, necessitating monitoring resistance to antimalarial agents. However, since its first report in 1989, the burden of drug-resistant *P. vivax* is still unclear and its underlying mechanism, epidemiology and drug efficacy have not been well characterized [17]. Four main methods, in vivo therapeutic efficacy studies, in vitro assay, drug concentration measurement, as well as molecular markers analysis, are used to monitor antimalarial drug efficacy and resistance. Among these, molecular markers are widely preferred due to their practical advantages over in vivo and in vitro tests. Molecular markers allow population-level screening, and samples on filter paper are easily obtained, transported and stored, thus avoiding host confounding factors [22]. The epidemiology of drug resistance of *P. vivax* varies across the GMS, hence molecular marker surveillance is encouraged to inform local drug policy.

In the present study, the prevalence of *Pvdhfr* mutation type, including point mutation and mutant tandem repeat, was high (94.83%, 55/58), which was similar to the reports in southern Thailand and western Myanmar. However, one study in Xishuangbanna Prefecture of Yunnan Province (southern Yunnan, bordering Myanmar in the west and Laos and Vietnam in the south) between 2009 and 2010, and another in India between 2005 and 2007, found that prevalence of *Pvdhfr* was lower than in our study [16] [17, 23, 24]. Furthermore, point mutations at codons 57, 58, 61 and 117 in the *Pvdhfr* gene were detected in 27.59–48.28% of isolates in the current study. These results were lower than those in southern Thailand and western Myanmar, but higher than those in Xishuangbanna of Yunnan and India [16, 17, 23, 24]. Regarding the patterns of mutation types, single and double mutants were the dominant genotypes in western Yunnan in our study, while quadruple mutation was the most common in Myanmar, Thailand and southern Yunnan [16, 17, 23, 25]. Previous studies identified that mutations at residues 117 and 58 arose first under drug pressure, so they were more highly mutated than others [26]. These results confirmed that the mutation types at codons 117 and 58 were the most frequent. Triple and quadruple mutations were more associated with high level of SP resistance than double or single mutations were. Our study indicated that *P. vivax* in western Yunnan might be under stronger drug pressure than those in western Myanmar and southern Thailand [27].

Mutant tandem repeats are also suggested to be associated with *P. vivax* antifolate resistance, and the frequency of Type II (H99S type) and Type III (deletion type) was 32.75 and 37.93%, respectively. This was consistent with a previous studies that reported that most isolates in India and Cambodia were deletion type [24, 28]. Nevertheless, the highest frequency of tandem repeat variants was for wild type in southern Thailand and Xishuangbanna Prefecture, Yunnan [16, 23]. In Anhui Province (Central China), Type II (H99S type) was the most common [16].

Similar to *Pvdhfr*, the frequency of mutant *Pvdhps*, especially the highly mutant types (triple or quadruple types), was less than that in southern Thailand and southern Myanmar [21, 23]. In addition, compared with another border region, Xishuangbannan of Yunnan, *Pvdhps* in our sampling region was more conserved, with higher proportions of wild type and fewer highly mutated types, although the isolates from Xishuangbannan were collected nearly 10 years ago [16]. Considering the similar drug policy in this study area and Xishuangbannan, it is still unclear whether the disagreement resulted from spatial heterogeneity or drug susceptibility to sulfadoxine.and as such, further study is required.

Several studies have provided evidence that *Pvmdr1* mutations are associated with reduced susceptibility to CQ [17, 29, 30]. Therefore, *Pvmdr1* is considered to be a strong candidate marker of drug resistance [23]. The prevalence of *Pvmdr1* T958M and F1076L mutations in our study was consistent with previous studies, showing that T958F was harbored in all the isolates and F1076L was in most of them [13, 21]. However, no Y976F was found in our study, while it was frequently reported with considerable prevalence in different endemic areas, including Indonesia, Thailand, Cambodia, India, Papua New Guinea and Ethiopia [29–35]. This is not surprising as 98.51% of patients were categorized as having an adequate clinical and parasitological response to CQ by an in vivo therapeutic efficacy study in Yingjiang and Tengchong, Yunnan [36]. Our study indicated that *Pvmdr1* at codon 976 was conserved in this area, although this needs to be confirmed.

The possible role of *Pvcrt-o* in CQ resistance is controversial. Several studies have found a negative link between K10 insertion and reduced CQ IC_50_, while others have shown that *Pvcrt-o* expression decreased susceptibility to CQ by 2.2-fold [30, 37]. The K10 *Pvcrt-o* gene insertion was found in 18.97% isolates in our study, which was less than in previous studies in Myanmar that reported 46.15% in Yangon in 1999, 72.73% in Shwegyin, 66.67% in Kawthaung and 48.33% in Buthidaung between 2009 and 2016 [17, 35]. Conversely, K10 insertion was rarely observed in Thailand from 2012 to 2018, or from the Thailand–Myanmar border or Thailand–Cambodia border in 2008 or 2014 [21, 23]. Given the geographical genetic differences among parasite populations from the GMS, the prevalence of K10 insertion in *Pvcrt-o* in the current and previous studies showed significant temporal and spatial heterogeneity [38]. The discrepancy may have resulted from differences in study sites or sample size.

## Conclusions

In conclusion, the present study demonstrated the prevalence and molecular pattern of candidate drug resistance markers *Pvdhfr*, *Pvdhps*, *Pvmdr1* and *Pvcrt-o* of imported *P. vivax* cases to Yingjiang county in Western Yunnan, along the China–Myanmar border. Diversity of molecular patterns of resistance markers *Pvdhfr*, *Pvdhps*, *Pvmdr1* and *Pvcrt-o* was found. This study helped to provide evidence for drug policy update.

## Supplementary information

**Additional file 1 Table S1** Primers and cycling conditions for *Pvcrt-o, Pvmdr1, Pvdhps* and *Pvdhfr* genotyping assay. **Table S2** Combined analysis of all mutations from *P. vivax* isolates.

## Data Availability

The datasets analyzed in this study are available from the corresponding author on reasonable request.

## Abbreviations

GMS: Greater Mekong subregion
WHO: World Health Organization
CQ: Chloroquine
IC_50_: Inhibitory concentration 50
