# Supplementary material for: Prevalence of molecular markers associated with drug resistance of Plasmodium vivax isolates in Western Yunnan Province, China
Source: BMC Infect Dis. 2020 Apr 25;20:307. doi: 10.1186/s12879-020-05032-4 (PMC7183581; doi:10.1186/s12879-020-05032-4)
Supplement: Supplementary file 1 — Additional file 1 Table S1 Primers and cycling conditions for Pvcrt-o, Pvmdr1, Pvdhps and Pvdhfr genotyping assay. Table S2 Combined analysis of all mutations from P. vivax isolates. [file 12879_2020_5032_MOESM1_ESM.docx]

Table S1 Primers and cycling conditions for *Pvcrt-o, Pvmdr1, Pvdhps* and *Pvdhfr* genotyping assay

| Genes | Sequences (5’→3’) | Cycling conditions | Product size(bp) |
| --- | --- | --- | --- |
| *Pvcrt-o* | F: TCC TTG CCG CTG ATT CTA CG  R: GGT AAC GTT CAT CGG GGG TT | 95°C 10 min, [95 °C 30 s, 60 °C 45 s, 72 °C 1 min]×35 cycles, 72 °C 10 min | 327 |
| *Pvmdr1*(1st) | F: ACG ACA TGA TCC AAA CGA CA  R: CTT ATA TAC GCC GTC CTG CAC | 94 °C 5 min, [94 °C 30 s, 60 °C 30 s, 68 °C 3 min]×20 cycles, 68 °C 5 min | 2784 |
| *Pvmdr1*(2nd) | F: GGA TAG TCA TGC CCC AGG ATT G  R: CAT CAA CTT CCC GGC GTA GC | 94 °C 5 min, [94 °C 30 s, 60 °C 30 s, 68 °C 3 min]×40 cycles, 68 °C 5 min | 604 |
| *Pvdhps*(1st) | F: GAT GGC GGT TTA TTT GTC G  R: GCT GAT CTT TGT CTT GAC G | 94 °C 5 min, [94 °C 30 s, 58 °C 30 s, 68 °C 1 min]×20 cycles, 68 °C 5 min | 979 |
| *Pvdhps* (2nd) | F: GCT GTG GAG AGG ATG TTC  R: CCG CTC ATC AGT CTG CAC | 94 °C 5 min, [94 °C 30 s, 58 °C 30 s, 68 °C 45 s]×40 cycles, 68 °C 5 min | 755 |
| *Pvdhfr*(1st) | F: CAC CGC ACC AGT TGA TTC CT  R: CCT CGG CGT TGT TCT TCT | 94 °C 5 min, [94 °C 30 s, 58 °C 30 s, 68 °C 1 min]×20 cycles, 68 °C 5 min | 1009 |
| *Pvdhfr* (2nd) | F: CCC CAC CAC ATA ACG AAG  R: CCC CAC CTT GCT GTA AAC C | 94 °C 5 min, [94 °C 30 s, 58 °C 30 s, 68 °C 45 s]×40 cycles, 68 °C 5 min | 731 |

Table S2 Combined analysis of all mutations from *P. vivax* isolates

| Haplotypes | *Pvcrt-o* | *Pvdhfr* | | | | | | | *Pvmdr1* | | | | *Pvdhps* | | | | | | No. of isolates |
| --- | --- | --- | --- | --- | --- | --- | --- | --- | --- | --- | --- | --- | --- | --- | --- | --- | --- | --- | --- |
|  | AAG insert | I13 | F57L/I | S58R | T61M | H99S | S117T/N | I173 | T958M | Y976F | K997 | F1076L | S382A | A383G | K512M | A553G | R580 | V585 |  |
| 1 | Mutant | I | I | R | M | H | T | I | M | Y | K | F | S | G | K | G | R | V | 1 |
| 2 | Mutant | I | F | R | T | / | N | I | M | Y | K | F | S | A | K | A | R | V | 1 |
| 3 | Mutant | I | F | S | T | / | S | I | M | Y | K | L | S | A | K | A | R | V | 1 |
| 4 | Wild | I | F | S | T | / | S | I | M | Y | K | L | S | G | K | A | R | V | 1 |
| 5 | Wild | I | F | S | T | S | S | I | M | Y | K | L | S | A | K | A | R | V | 3 |
| 6 | Wild | I | F | S | T | S | S | I | M | Y | K | L | S | G | K | A | R | V | 14 |
| 7 | Wild | I | I | R | M | H | T | I | M | Y | K | F | S | A | K | A | R | V | 2 |
| 8 | Wild | I | I | R | M | H | T | I | M | Y | K | L | S | A | K | A | R | V | 2 |
| 9 | Mutant | I | I | R | M | H | T | I | M | Y | K | L | S | A | K | A | R | V | 1 |
| 10 | Wild | I | I | R | M | H | T | I | M | Y | K | L | S | G | K | G | R | V | 2 |
| 11 | Mutant | I | I | R | M | H | T | I | M | Y | K | L | S | G | K | G | R | V | 1 |
| 12 | Mutant | I | F | R | T | / | N | I | M | Y | K | F | S | G | K | G | R | V | 1 |
| 13 | Wild | I | I | R | M | S | T | I | M | Y | K | L | S | G | K | A | R | V | 1 |
| 14 | Mutant | I | I | R | M | S | T | I | M | Y | K | L | S | G | K | G | R | V | 1 |
| 15 | Mutant | I | L | R | M | H | T | I | M | Y | K | F | C | G | E | G | R | V | 1 |
| 16 | Wild | I | L | R | M | H | T | I | M | Y | K | F | S | A | K | A | R | V | 2 |
| 17 | Wild | I | L | R | M | H | T | I | M | Y | K | F | S | G | K | G | R | V | 1 |
| 18 | Mutant | I | L | R | M | H | T | I | M | Y | K | L | S | G | K | A | R | V | 1 |
| 19 | Wild | I | F | R | T | / | N | I | M | Y | K | L | S | A | K | A | R | V | 8 |
| 20 | Wild | I | F | R | T | / | N | I | M | Y | K | L | S | G | K | A | R | V | 1 |
| 21 | Mutant | I | F | R | T | / | N | I | M | Y | K | L | S | G | K | G | R | V | 1 |
| 22 | Wild | I | F | S | T | H | S | I | M | Y | K | L | S | A | K | A | R | V | 1 |
| 23 | Wild | I | F | S | T | H | S | I | M | Y | K | L | S | G | K | A | R | V | 1 |
| 24 | Wild | I | F | S | T | / | S | I | M | Y | K | L | S | A | K | A | R | V | 8 |
| 25 | Mutant | I | F | S | T | H | S | I | M | Y | K | L | S | G | K | A | R | V | 1 |
